# Enhancing intracranial aneurysm rupture risk prediction with a novel multivariable logistic regression model incorporating high-resolution vessel wall imaging

**DOI:** 10.3389/fneur.2024.1507082

**Published:** 2025-01-07

**Authors:** Zihang Wang, Chang Yan, Wenqing Yuan, Shuangyan Jiang, Yongxiang Jiang, Ting Chen

**Affiliations:** ^1^Department of Radiology, The Second Affiliated Hospital of Chongqing Medical University, Chongqing, China; ^2^The Second Clinical College, Chongqing Medical University, Chongqing, China; ^3^Department of Neurosurgery, The Second Affiliated Hospital of Chongqing Medical University, Chongqing, China

**Keywords:** intracranial aneurysm, parent artery, HR-VWI, PHASES score, ELAPSS score

## Abstract

**Objective:**

This study aimed to develop and validate a multivariate logistic regression model for predicting intracranial aneurysm (IA) rupture by integrating clinical data, aneurysm morphology, and parent artery characteristics using high-resolution vessel wall imaging (HR-VWI).

**Methods:**

A retrospective analysis was conducted on 298 patients with 386 aneurysms. Patients were randomly divided into training (*n* = 308) and validation (*n* = 78) sets. Key predictors, including aneurysm size, shape, aneurysm wall and parent artery wall enhancement, were identified through univariate analysis and then used to build the prediction model using multivariate logistic regression. The model was visualized as a nomogram and compared to PHASES and ELAPSS scores.

**Results:**

The logistic regression model demonstrated superior predictive performance with an area under the curve of 0.814, which was significantly higher than PHASES and ELAPSS scores (*p* < 0.05). The model revealed strong calibration and good agreement between predicted and observed rupture probabilities.

**Conclusion:**

The multivariate model based on HR-VWI, which incorporates aneurysm and parent artery features, provides a more accurate prediction of IA rupture risk than conventional scoring systems, offering a valuable tool for clinical decision-making.

## 1 Introduction

Intracranial aneurysms (IAs) are common vascular conditions that can lead to subarachnoid hemorrhage (SAH), a severe form of stroke with a fatality rate approaching 50% (1, 2). Accurate evaluation of rupture risk is crucial for clinical decision-making. Traditional models, such as PHASES and ELAPSS scores, only rely on clinical and morphological data, resulting in moderate predictive accuracy (3–6).

High-resolution vessel wall imaging (HR-VWI) surpasses conventional vascular imaging by providing detailed insight into aneurysm architecture and wall enhancement (AWE) (7). Studies suggest that AWE, which reflects inflammatory activity after contrast administration, strongly correlates with aneurysm rupture risk, thus improving the accuracy of IA risk predictions (8). This technique is more effective for assessing the likelihood of IA rupture. Current research using HR-VWI for IA rupture risk primarily focuses on qualitative and quantitative assessments of AWE (9, 10), often neglecting the role of the parent artery. However, emerging evidence indicates that vessel wall enhancement near the aneurysm neck is associated with aneurysm development and progression (11). Despite this, few studies have incorporated parent artery analysis in HR-VWI-based rupture risk assessments, underutilizing its predictive potential.

This study aimed to improve IA rupture risk prediction by integrating clinical profiles, aneurysm and parent artery morphometrics, and HR-VWI-derived features. We developed a predictive model using multivariate logistic regression, visualized it as a nomogram, and compared its performance against PHASES and ELAPSS scoring systems.

## 2 Materials and methods

### 2.1 Study subjects

This study was approved by our institutional review committee (Ethics Committee: Ke Lun Review No. 194) and obtained written informed consent from all participants. We conducted a retrospective analysis of patients diagnosed with IAs through head computed tomography angiography at our hospital between October 2019 and October 2023. The inclusion criteria were as follows: (1) ruptured or unruptured saccular aneurysms with SAH attributable to aneurysm rupture; (2) high-quality three-dimensional (3D) HR-VWI data indicating the aneurysm and adjacent 3 mm of the parent artery. The exclusion criteria were as follows: (1) incomplete HR-VWI images; (2) dissection or fusiform aneurysms; (3) post-treatment aneurysms; (4) multiple aneurysms with unclear rupture source; (5) poor HR-VWI image quality or aneurysms too small to assess wall features.

### 2.2 Data collection

#### 2.2.1 Patient information

We collected data on age, gender, ethnicity, and previous medical history (including hypertension, diabetes, hyperlipidemia, coronary heart disease, smoking, and history of SAH). We also recorded the history of the present illness and the date of imaging, as obtained from the hospital’s electronic medical records for both inpatients and outpatients. For incomplete records, supplementary information was gathered through telephone follow-up.

#### 2.2.2 Risk scoring

The PHASES score (12) was used to assess aneurysm rupture risk based on ethnicity, hypertension, age, aneurysm size, history of SAH, and location. The ELAPSS score (13) was applied to evaluate the growth risk of IA, considering the history of SAH, aneurysm location, age, ethnicity, aneurysm size, and shape.

### 2.3 Imaging examination

#### 2.3.1 Magnetic resonance imaging (MRI) protocol

MRI scans were conducted using an Ingenia CX 3.0 T scanner with a 32-channel head coil. The protocol began with 3D time-of-flight (TOF) magnetic resonance angiography (MRA) for IA localization, followed by axial 3D T1-weighted volume isotropic turbo spin echo acquisition (VISTA) sequences for targeted imaging of the parent artery. A contrast agent, gadopentetate dimeglumine (0.1 mmol/kg, Gd-DOTA, Jiangsu, China), was manually injected into the antecubital vein. HR-VWI was repeated 5 min after the contrast injection to acquire contrast-enhanced HR-VWI (CE-HR-VWI). Scan parameters included a field of view (FOV) 200 × 200 mm^2^, slice thickness 0.50 mm, slices 80, TR/TE 800/22 ms, flip angle 90°, and voxel size 0.6 × 0.6 × 0.6 mm^3^. Images were processed using the Philips IntelliSpace Portal V12 workstation.

### 2.4 Imaging assessment

#### 2.4.1 Morphological features of IAs and parent arteries

**Figure 1:**
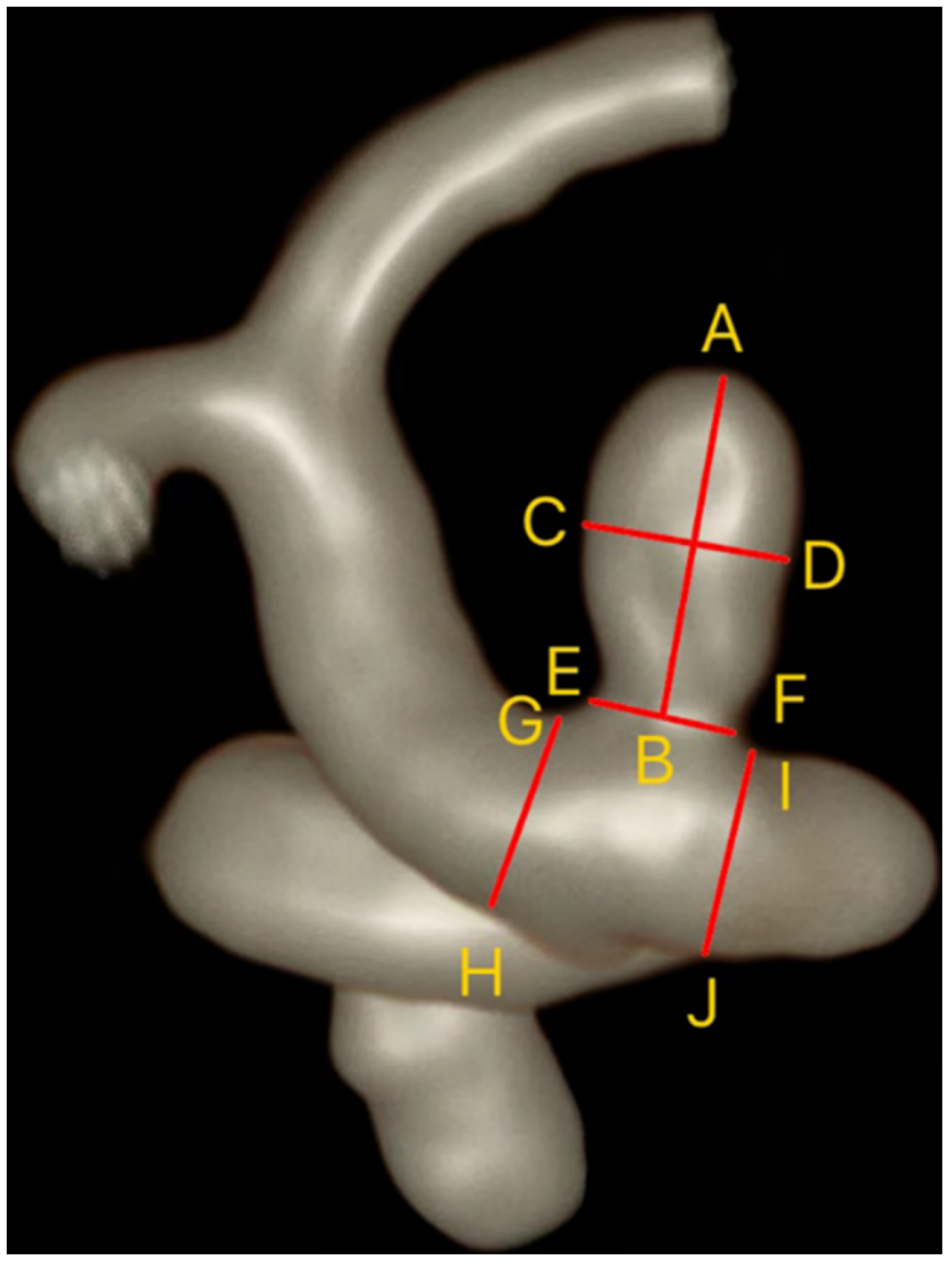
Measurement of IAs and parent artery. Maximum height (MH) = AB; Maximum width (MW) = CD; Neck width (NW) = EF; Parent artery diameter (PAD) = (GH + IJ)/2.

We evaluated the location, configuration (whether at bifurcations or sidewalls), and shape (including regularity and the presence of daughter sacs) of aneurysms using three-dimensional reconstructed images from TOF-MRA. Using two-dimensional measurement tools on HR-VWI images, we obtained the size of aneurysms, including neck width (NW), maximum height (MH), maximum width (MW), and parent artery diameter (PAD) (Figure 1) (14). From these measurements, we calculated the aneurysm’s aspect ratio (AR), size ratio (SR), and neck-to-parent ratio (NPR).

#### 2.4.2 Characteristics of aneurysm and parent artery walls on HR-VWI

Two neuroradiologists with over 10 years of diagnostic experience independently performed a blinded analysis of the HR-VWI and CE-HR-VWI images. The two neuroradiologists were unaware of the clinical data but were aware of the location of the aneurysm. When there was a disagreement, a third neuroradiologist with over 20 years of diagnostic experience would intervene to negotiate a resolution.

##### 2.4.2.1 Qualitative analysis

**Figure 2:**
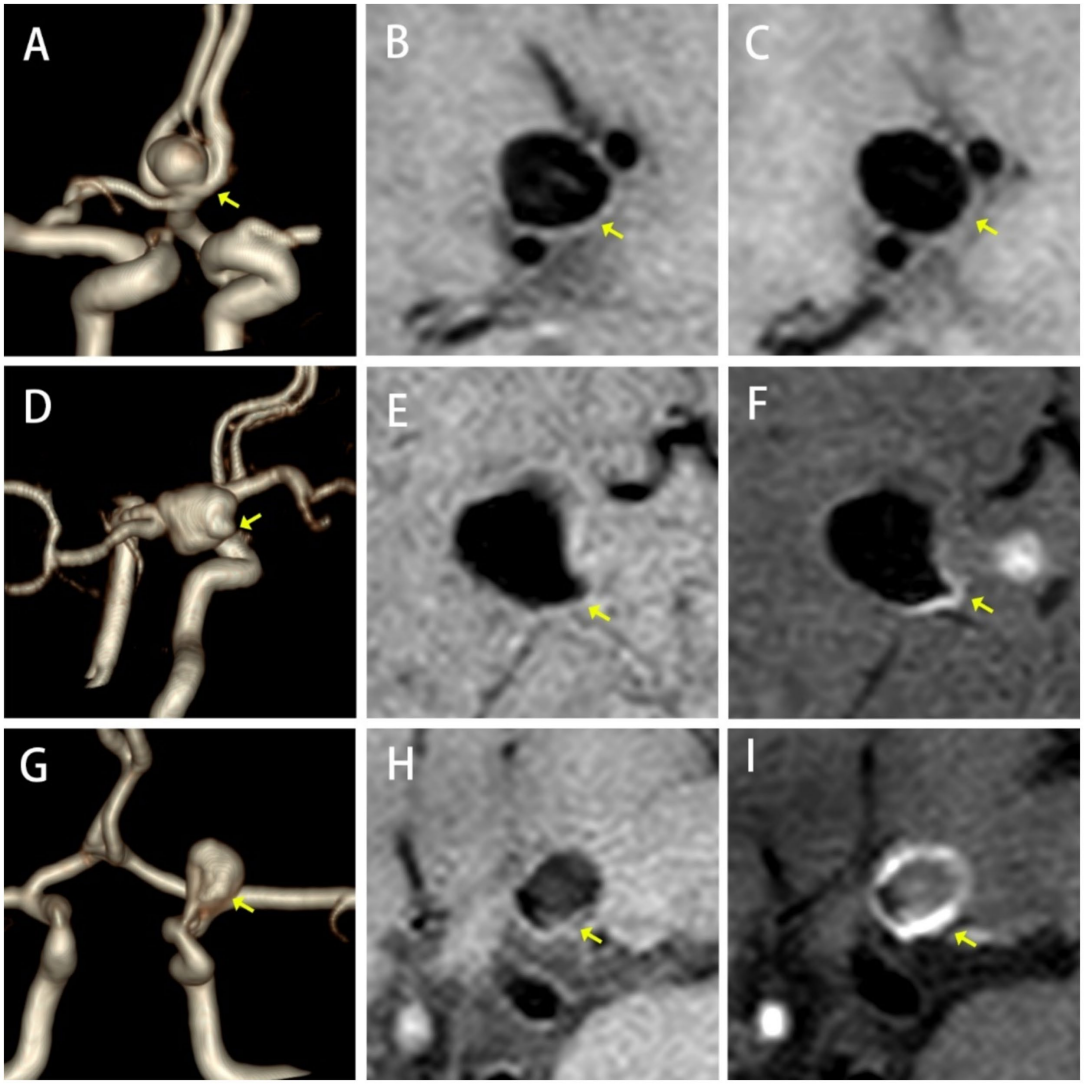
Types of AWE. No Enhancement, **(A)** TOF-MRA, **(B)** HR-VWI, and **(C)** CE-HR-VWI; Focal Enhancement, **(D)** 3D-TOF-MRA, **(E)** HR-VWI, and **(F)** CE-HR-VWI; Ring Enhancement, **(G)** 3D-TOF-MRA, **(H)** HR-VWI, and **(I)** CE-HR-VWI.

A qualitative analysis was conducted to evaluate AWE severity, which was categorized into three grades: Grade 0 (no enhancement, similar signal intensity on both images), Grade 1 (focal, localized non-circular enhancement), and Grade 2 (ring enhancement, with enhancement throughout the entire aneurysm wall) (15) (Figure 2).

##### 2.4.2.2 Quantitative analysis

Two neuroradiologists conducted quantitative measurements evaluating the following parameters from the optimal viewing angle (sagittal, frontal plane, and horizontal plane). All measurements were made at an image magnification of 500%.

**Figure 3:**
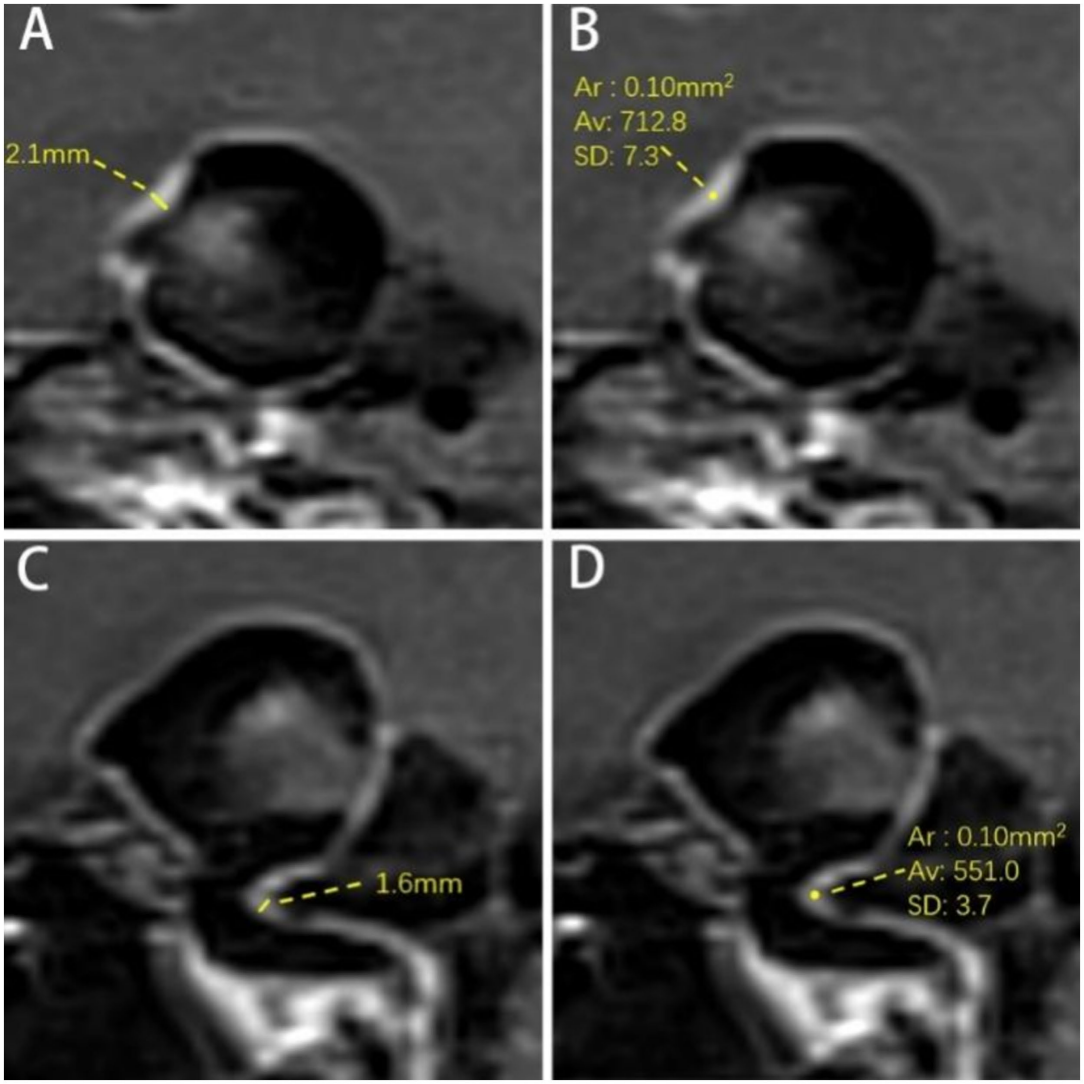
Measurement of aneurysm wall and parent artery. **(A)** Measurement of AWT. The AWT is 2.1 mm. **(B)** Measurement SI of AWE. The signal intensity of the aneurysm wall is 712.8. **(C)** Parent artery wall thickness (PAWT). The PAWT is 1.6 mm. **(D)** Parent artery wall signal intensity. The signal intensity of the parent artery wall is 551.0. Ar = Average Range; Av = Average Signal Intensity of ROI; SD = Standard Deviation.

The thickest part of the aneurysm wall and the parent artery wall were measured three times at the direct boundary, and the average value was calculated as aneurysm wall thickness (AWT) and parent artery wall thickness (PAWT) (Figures 3A,C).

AWE was evaluated using the Wall Enhancement Index (WEI) and the contrast ratio between the aneurysm wall and the pituitary stalk (CR_stalk_) (Equations 1, 2) (16, 17). Parent artery wall enhancement (PAWE) was evaluated using the parent artery wall enhancement index (PWEI) and the contrast ratio between the parent artery wall and the pituitary stalk (PCR_stalk_) (Equations 3, 4).


(1) $$WEI=\;\frac{SI_{\mathrm{AW}\left(\mathrm{Post}\right)}/SI_{\mathrm{RFWM}\left(\mathrm{Post}\right)}-SI_{\mathrm{AW}\left(Pre\right)}/SI_{\mathrm{RFWM}\left(Pre\right)}}{\;SI_{\mathrm{AW}\left(Pre\right)}/SI_{\mathrm{RFWM}\left(Pre\right)}}$$

(2) $$CR_{\mathrm{stalk}}=\;\frac{SI_{\mathrm{AW}\left(\mathrm{Post}\right)}}{\;SI_{PS\left(\mathrm{Post}\right)}}$$

(3) $$\mathrm{PWEI}=\;\frac{SI_{PA\left(\mathrm{post}\right)}/SI_{\mathrm{RFWM}\left(\mathrm{Post}\right)}-SI_{PA\left(pre\right)}/SI_{\mathrm{RFWM}\left(Pre\right)}}{\;SI_{PA\left(pre\right)}/SI_{\mathrm{RFWM}\left(Pre\right)}}$$

(4) $$PCR_{\mathrm{stalk}}=\;\frac{SI_{PA\left(\mathrm{post}\right)}}{\;SI_{PS\left(\mathrm{post}\right)}}$$

The post-contrast SI of the aneurysm wall *(SI_AW(post)_)* and parent artery (*SI_PA(post)_*) was measured separately three times on post-contrast images on the layer of their respective maximum diameters, and the highest value was recorded as the enhanced SI (Figures 3B,C). Furthermore, the measurement range for parent artery was within 3 mm of the aneurysm neck. The region of interest (ROI) was 0.1 mm^2^. The pre-contrast SI of the aneurysm wall (*SI_AW(pre)_*) and parent artery (*SI_PA(pre)_*) was measured using the same method at the corresponding site. The SI of the right frontal white matter (*SI_RFWM_*) was measured as a reference on both pre- and post-contrast images. The ROI was 20 mm^2^. Additionally, the pituitary stalk SI (*SI_PS(post)_*) was measured three times on the upper, middle, and lower parts of the pituitary stalk on CE-HR-VWI images and the highest value among them was recorded. The ROI was 0.1 mm^2^ (18).

### 2.5 Statistical methods

Statistical analysis was performed using R software (version 4.2.3). Kappa statistics evaluated inter-rater agreement on AWE (*κ* > 0.75, indicating good concordance). Intra-class correlation coefficient (ICC) was used to assess the reliability for WEI, PWEI, CR_stalk_, PCR_stalk_, AWT, and PAWT (ICC > 0.75, indicating good reliability). The Shapiro–Wilk test was applied to assess the distributions of variables. T-tests, F-tests, and Chi-square tests were used to differentiate ruptured and unruptured IAs. Multivariate logistic regression including univariate predictors (*p* < 0.2) attained aneurysm rupture with 95% confidence interval [CI] and then selected predictors (*p* < 0.05) for model construction. Receiver-operating characteristic (ROC) curves were used to benchmark model accuracy, and the PHASES and ELAPSS normalized scores (0–1) were compared with the logistic regression model. The DeLong test (*p* < 0.05) was applied to determine statistical significance (19).

## 3 Results

### 3.1 Univariate and multivariate analyses of IA rupture prediction factors

**Figure 4:**
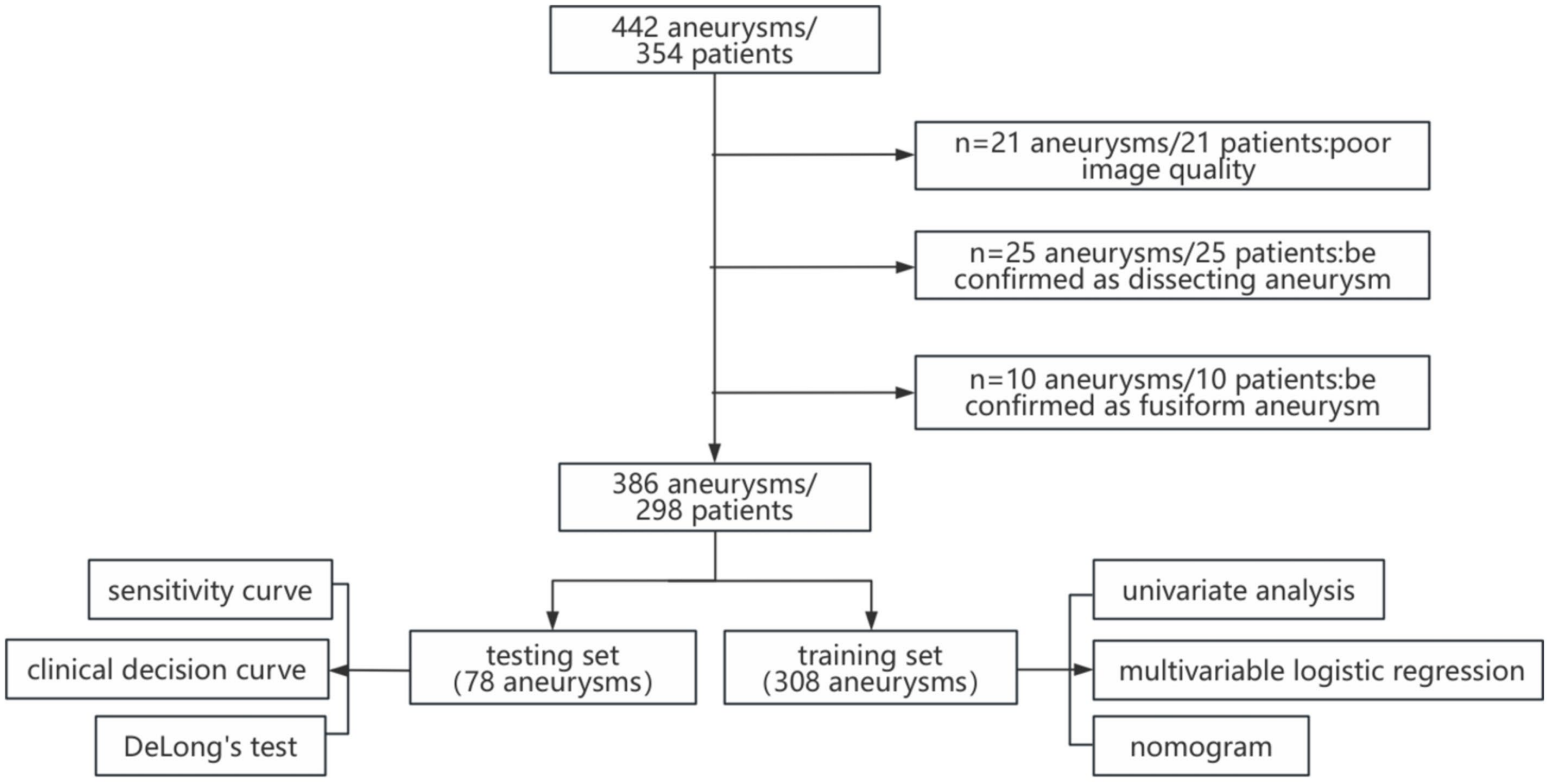
Flowchart of research.

A total of 386 IAs from 298 patients were randomly divided into training (80%) and validation (20%) sets to analyze predictors of IA rupture and for model development and validation (Table 1; Figure 4). In both sets, IAs were classified into two groups: ruptured IAs were classified as the ruptured group, and unruptured IAs were classified as the unruptured group.

**Table 1:** Characteristics of patients and aneurysms in training and testing sets.

Features	Groups	
	Training set (*n* = 308)	Testing set (*n* = 78)
I Clinical features		
Mean Age (years)	60.0 [51.0, 68.0]	57.5 [51.0, 70.0]
Male/Female (%)	210/98 (68.7%/31.3%)	49/29 (62.8%/37.2%)
History of prior SAH (%)	3 (0.97%)	2 (2.56%)
Hypertension (%)	158 (51.3%)	49 (62.8%)
Hyperlipidemia (%)	112 (36.4%)	34 (43.6%)
Coronary artery disease (%)	34 (11.0%)	5 (6.41%)
Diabetes (%)	34 (11.0%)	9 (11.5%)
Smoking History (%)	71 (23.1%)	20 (25.6%)
II Morphological features		
Aneurysm shape		
Irregular	195 (63.3%)	32 (41.0%)
Regular	113 (36.7%)	46 (59.0%)
Daughter sacs		
Present	50 (16.2%)	14 (17.9%)
Absent	258 (83.8%)	64 (82.1%)
Aneurysm location		
Internal carotid artery	170 (55.2%)	27 (34.6%)
Anterior cerebral artery	11 (3.57%)	8 (10.3%)
Anterior communicating artery	25 (8.12%)	8 (10.3%)
Middle cerebral artery	55 (17.9%)	15 (19.2%)
Posterior circulation	18 (5.84%)	8 (10.3%)
Posterior communicating artery	29 (9.42%)	12 (15.4%)
Side Wall/Bifurcation (%)	259/49 (84.1%/15.9%)	60/18 (76.9%/23.1%)
Aneurysm size (mm)		
NW	3.32 [2.60, 4.35]	3.48 [2.27, 4. 34]
MW	3.72 [2.65, 5.81]	3.97 [2.61, 6.72]
MH	3.74 [2.64, 5.70]	3. 59 [2.66, 5.79]
PAD (mm)	3.09 [2.34, 3.76]	2.79 [2.14, 3.43]
Morphological ratio		
AR	1.18 [0.91, 1.56]	1.20 [0.88, 1.69]
SR	1.28 [0.81, 2.05]	1.41 [0.83, 2.78]
NPR	1.08 [0.81, 1.59]	1.18 [0.83, 1.76]
III Aneurysm wall features		
Thickness	0.96 [0.78, 1.13]	1.00 [0.80, 1.18]
Thickness classification		
<1 mm	176 (57.1%)	39 (50.0%)
≥1 mm	132 (42.9%)	39 (50.0%)
Enhancement classification		
No enhancement	184 (59.7%)	33 (42. 3%)
Focal enhancement	40 (13.0%)	11 (14.1%)
Ring enhancement	84 (27.3%)	34 (43.6%)
WEI	0.40 [0.26, 0.82]	0. 61 [0.30, 1.03]
CR_stalk_	0.47 [0.38, 0.72]	0.59 [0.40, 0.80]
IV Parent artery wall features		
Thickness	0.92 [0.81, 1.05]	0.95 [0.85, 1.08]
PWEI	0.30 [0.17, 0.52]	0.35 [0.21, 0.56]
PCR_stalk_	0.39 [0.32; 0.54]	0.45 [0. 34, 0.59]
Atherosclerosis of the parent artery (%)	90 (29.2%)	20 (25.6%)
V Clinical risk score		
ELAPSS score	10.0 [5.00, 18.0]	11.0 [6.00, 18.0]
PHASES score	2.00 [1.00, 5.00]	4.00 [2.00, 5.00]

NW, Neck width; MW, Maximum width; MH, Maximum height; PAD, Parent artery diameter; AR, Aspect ratio; SR, Size ratio; NPR, Neck-to-parent ratio; WEI, Wall enhancement index; CR_stalk_, Contrast ratio of the aneurysm wall against the pituitary stalk; PWEI, Parent artery wall enhancement index; PCR_stalk_, Contrast ratio of the parent artery wall against the pituitary stalk.

Inter-rater reliability for AWE classification was high (*κ* = 0.871; 95% CI: 0.751–0.991). The consistency between the two evaluators in measuring morphological features and assessing enhancement SI assessments was also strong (ICC = 0.933; 95% CI: 0.886–0.961).

#### 3.1.1 Collection of prediction factors

In this study, 25 potential predictors were evaluated, covering clinical traits, morphological attributes of the aneurysm and its parent artery, and characteristics of both the aneurysm and parent artery walls. Specifically, clinical factors included age, gender, a history of hypertension, diabetes, hyperlipidemia, coronary artery disease, smoking, and prior SAH. Morphological parameters included aneurysm location, configuration (bifurcation or sidewall), morphology (regularity, presence of daughter sacs), size (NW, MH, and MW), and indices such as PAD, AR, SR, and NPR. Wall features were assessed using WEI, CR_stalk_, and AWT for the aneurysm wall, and PWEI, PCR_stalk_, and PAWT for the parent artery wall.

#### 3.1.2 Results of univariate analysis and multivariate logistic analysis

Univariate analysis identified 18 factors associated with aneurysm rupture, including (1) morphological characteristics: aneurysm location, shape (regularity, presence of daughter sacs), NW, MH, MW, PAD, AR, SR, NPR; (2) aneurysm wall characteristics: wall enhancement classification, parent artery thickness classification, WEI, CR_stalk_ and AWT; (3) parent artery wall characteristics: PWEI, PCR_stalk_ and PAWT (*p* < 0.05) (Table 2).

**Table 2:** Univariate analysis of IAs rupture prediction in patients with training set.

Features	Training set (*n* = 308)		*p*
	Unruptured group (*n* = 243)	Ruptured group (*n* = 65)	
I Clinical Features			
Mean age (years)	60.0 [51.0, 68.0]	57.0 [49.0, 67.0]	0.806
Male/Female (%)	167/76 (68.7%/31.3%)	43/22 (66.2%/33.8%)	0.222
History of Prior SAH (%)	3 (1.23%)	0 (0%)	1.0
Hypertension (%)	126 (51.9%)	32 (49.2%)	0.814
Hyperlipidemia (%)	89 (36.6%)	23 (35.4%)	0.968
Coronary artery disease (%)	30 (12.3%)	4 (6.15%)	0.233
Diabetes (%)	28 (11.5)%	6 (9.23%)	0.763
Smoking history (%)	57 (23.5%)	14 (21.5%)	0.873
II Morphological Features			
Aneurysm shape			
Irregular	68 (28.0%)	45 (69.2%)	<0.001
Regular	175 (72.0%)	20 (30.8%)	
Daughter sacs			
Present	22 (9.05%)	28 (43.1%)	<0.001
Absent	221 (90.95%)	37 (56.9%)	
Aneurysm location			
Internal carotid artery	147 (60.5%)	23 (35.4%)	<0.001
Anterior cerebral artery	9 (3.7%)	2 (3.08%)	
Anterior communicating artery	17 (7.0%)	8 (12.3%)	
Middle cerebral artery	44 (18.1%)	11 (16.9%)	
Posterior circulation	14 (5.76%)	4 (6.15%)	
Posterior communicating artery	12 (4.94%)	17 (26.2%)	
Side Wall/Bifurcation (%)	207/36 (85.2%/14.8%)	50/15 (76.9%/23.1%)	0.160
Aneurysm size (mm)			
NW	3.17 [2.50, 4.25]	3.61 [3.01, 4.60]	0.008
MW	3.50 [2.48, 5.65]	4.31 [3.30, 6.89]	0.001
MH	3.51 [2.44, 5.20]	5.00 [3.74, 6.46]	<0.001
PAD (mm)	3.12 [2.42, 3.81]	2.82 [2.24, 3.42]	0.047
Morphological ratio			
AR	1.13 [0.87, 1.44]	1.37 [1.17, 1.72]	<0.001
SR	1.12 [0.77, 1.86]	2.43 [1.44, 3.31]	0.004
NPR	1.03 [0.77, 1.52]	1.31 [1.06, 1.77]	<0.001
III Aneurysm wall features			
AWT	0.89 [0.72, 1.06]	1.13 [1.05, 1.29]	<0.001
Thickness classification			
<1 mm	163 (67.1%)	13 (20.0%)	<0.001
≥1 mm	80 (32.9%)	52 (80.0%)	
Enhancement classification			
No enhancement	170 (70.0%)	14 (21.5%)	<0.001
Focal enhancement	26 (10.7%)	14 (21.5%)	
Ring enhancement	47 (19.3%)	37 (57.0%)	
WEI	0.35 [0.23, 0.60]	0.91 [0.68, 1.46]	<0.001
CR_stalk_	0.42 [0.36, 0.57]	0.79 [0.67, 0.88]	<0.001
IV Parent artery wall features			
PAWT	0.89 [0.76, 1.00]	1.03 [0.94, 1.13]	<0.001
PWEI	0.26 [0.15, 0.39]	0.56 [0.46, 0.69]	<0.001
PCR_stalk_	0.36 [0.31, 0.46]	0.58 [0.49, 0.69]	<0.001
Atherosclerosis of the parent artery (%)	69 (28.4%)	21 (32.3%)	0.644
V Clinical risk score			
ELAPSS score	9.00 [5.00, 17.0]	15.0 [10.0, 20.0]	<0.001
PHASES score	2.00 [1.00, 5.00]	4.00 [2.00, 6.00]	0.007

NW, Neck width; MW, Maximum width; MH, Maximum height; PAD, Parent artery diameter; AR, Aspect ratio; SR, Size ratio; NPR, Neck-to-parent ratio; AWT, Aneurysm wall thickness; WEI, Wall enhancement index; CR_stalk_, Contrast ratio of the aneurysm wall against the pituitary stalk; PAWT, Parent artery wall thickness; PWEI, Parent artery wall enhancement index; PCR_stalk_, Contrast ratio of the parent artery wall against the pituitary stalk.

Multivariate analysis identified the following independent risk factors for IA rupture: irregular aneurysm shape (*p* = 0.004, odds ratio [OR] = 5.55, 95% CI: 1.72–17.89), MW of the aneurysm (*p* = 0.03, OR = 0.62, 95% CI: 0.40–0.96), CR_stalk_ (*p* = 0.01, OR = 107.21, 95% CI: 3.00–3828.00) and PCR_stalk_ (*p* = 0.007, OR = 189.0, 95% CI: 4.30–8309.76).

### 3.2 Construction and visualization of the multivariate logistic regression model

A multivariate logistic regression model (MLRM) was developed, incorporating four independent risk factors for IA rupture into a numerical assessment of rupture probability. The Equation is as follows:


(5) $$\begin{array}{l} \mathrm{LOG}\left[p\left(Αn\right)/1-p\left(Αn\right)\right]=1.945\times \mathrm{regularity}\left(0|1\right)\\ -0.303\times MW+5.440\times {CR}_{\mathrm{stalk}}+4.506\times {PCR}_{\mathrm{stalk}}-6.436 \end{array}$$

Equation 5 shows MLRM of IAs rupture risk.

**Figure 5:**
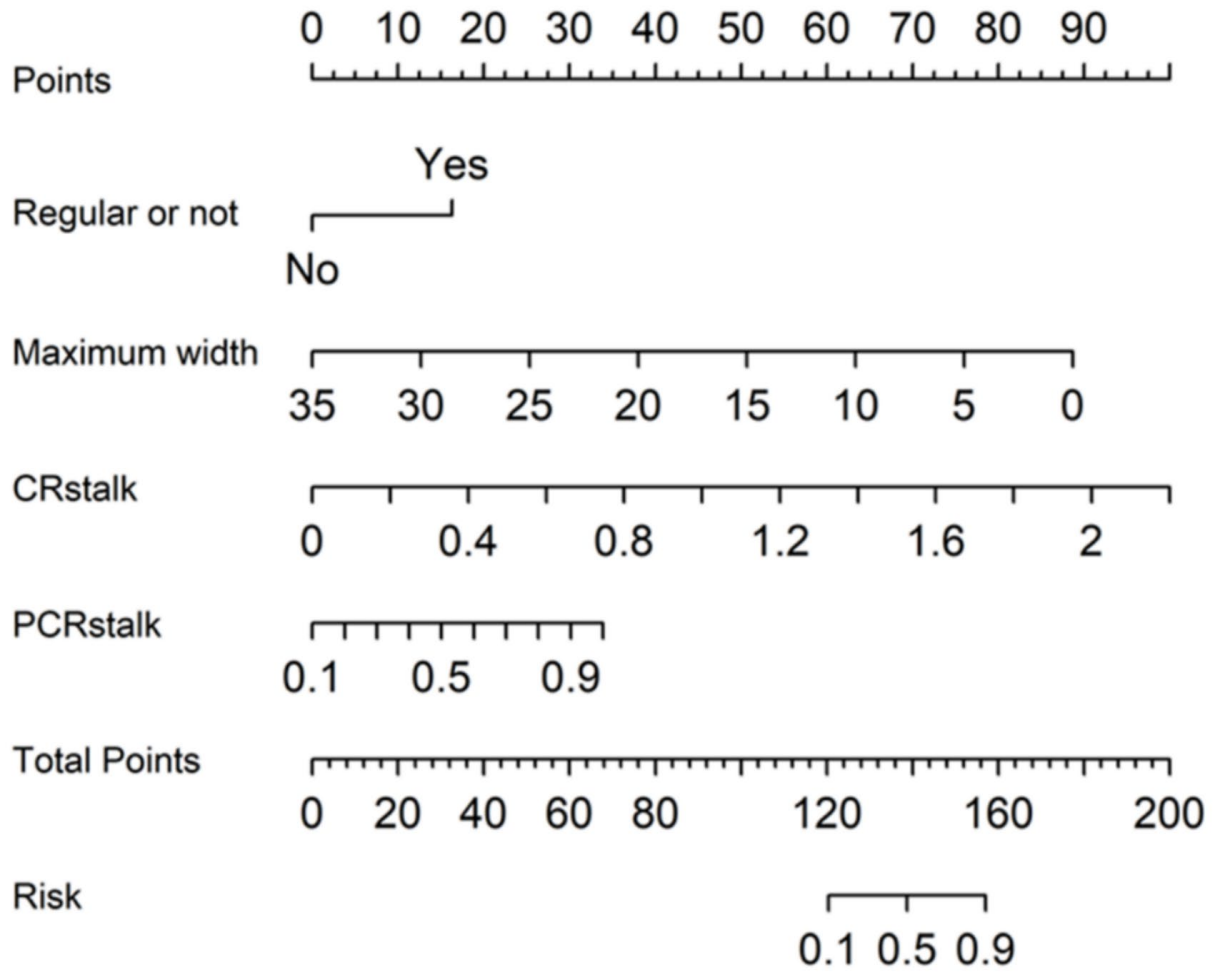
The nomogram of MLRM.

In this Equation, *p*(An) represents the estimated rupture probability of the aneurysm rupture. This predictive model has been converted into a visual nomogram (Figure 5).

### 3.3 Validation of the MLRM and comparison with ELAPSS and PHASES scores

**Figure 6:**
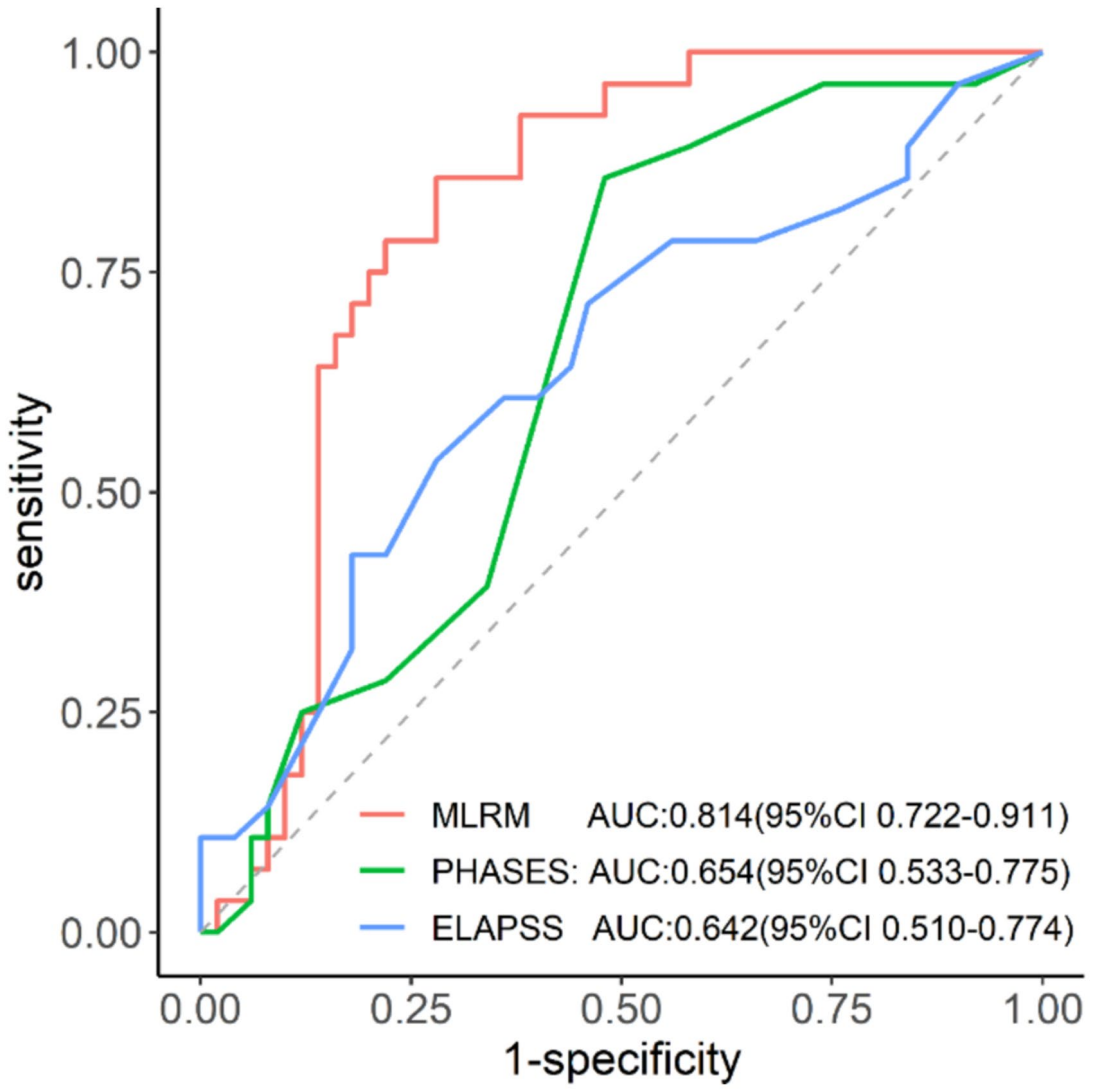
ROC-AUC analysis of MLRM, PHASES, and ELAPSS in the validation set.

The MLR nomogram model demonstrated strong predictive performance in the validation set, with an area under the ROC curve of 0.814 (95% CI: 0.722–0.911) (Figure 6), the highest among the three predictive models (Table 3). The DeLong test indicated a significant difference in predictive efficacy, with the MLR model outperforming the ELAPSS Score (*p* = 0.037) and the PHASES Score (*p* = 0.040).

**Table 3:** Comparison of the predictive efficiency of MLRM, PHASES, and ELAPSS.

Predictive efficiency	Nomogram	ELPSS score	PHASES score
AUC (95% CI)	0.814 (0.722–0.911)	0.642 (0.510–0.774)	0.654 (0.533–0.775)
cut-off (95% CI)	0.116 (0.083–0.141)	14.496 (11.224–18.367)	3.504 (2.159–4.286)
Sens (95%CI)	0.917 (0.788–1.000)	0.563 (0.381–0.750)	0.857 (0.728–0.987)
Spec (95%CI)	0.704 (0.583–0.831)	0.720 (0.596–0.844)	0.520 (0.382–0.658)
ACC (95%CI)	0.765 (0.761–0.770)	0.654 (0.648–0.660)	0.641 (0.635–0.647)
PPV (95%CI)	0.579 (0.425–0.732)	0.517 (0.335–0.699)	0.500 (0.359–0.641)
NPV (95%CI)	0.950 (0.857–1.000)	0.735 (0.611–0.858)	0.867 (0.745–0.988)

AUC, Area under the curve; Sens, Sensitivity; Spec, Specificity; ACC, Accuracy; PPV, Positive predictive value; NPV, negative predictive value.

**Figure 7:**
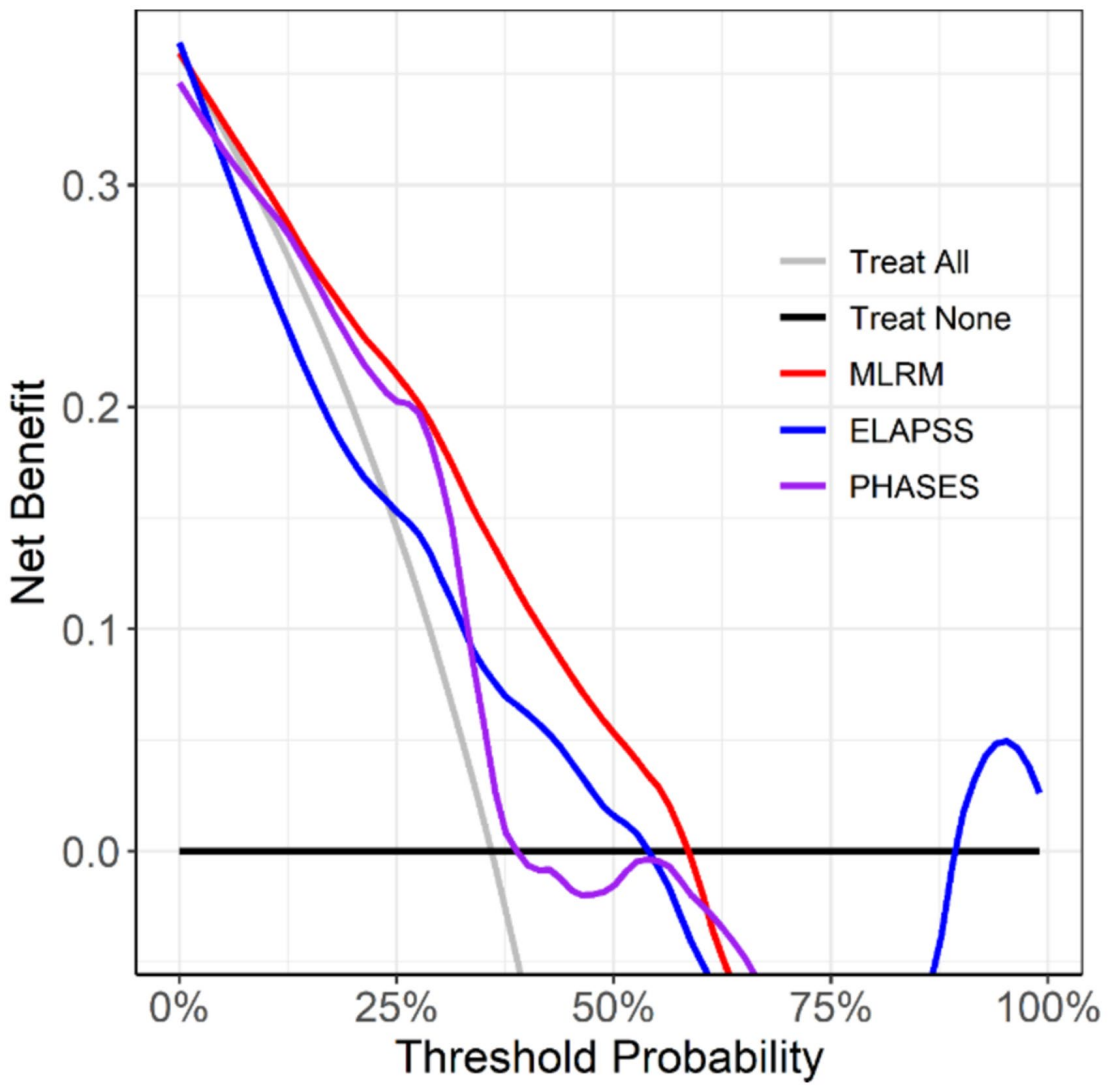
DCA curves of MLRM, PHASES, and ELAPSS.

The calibration curve (Figure 7) aligned closely with the diagonal line, suggesting good agreement between predicted and observed probabilities of IA rupture.

**Figure 8:**
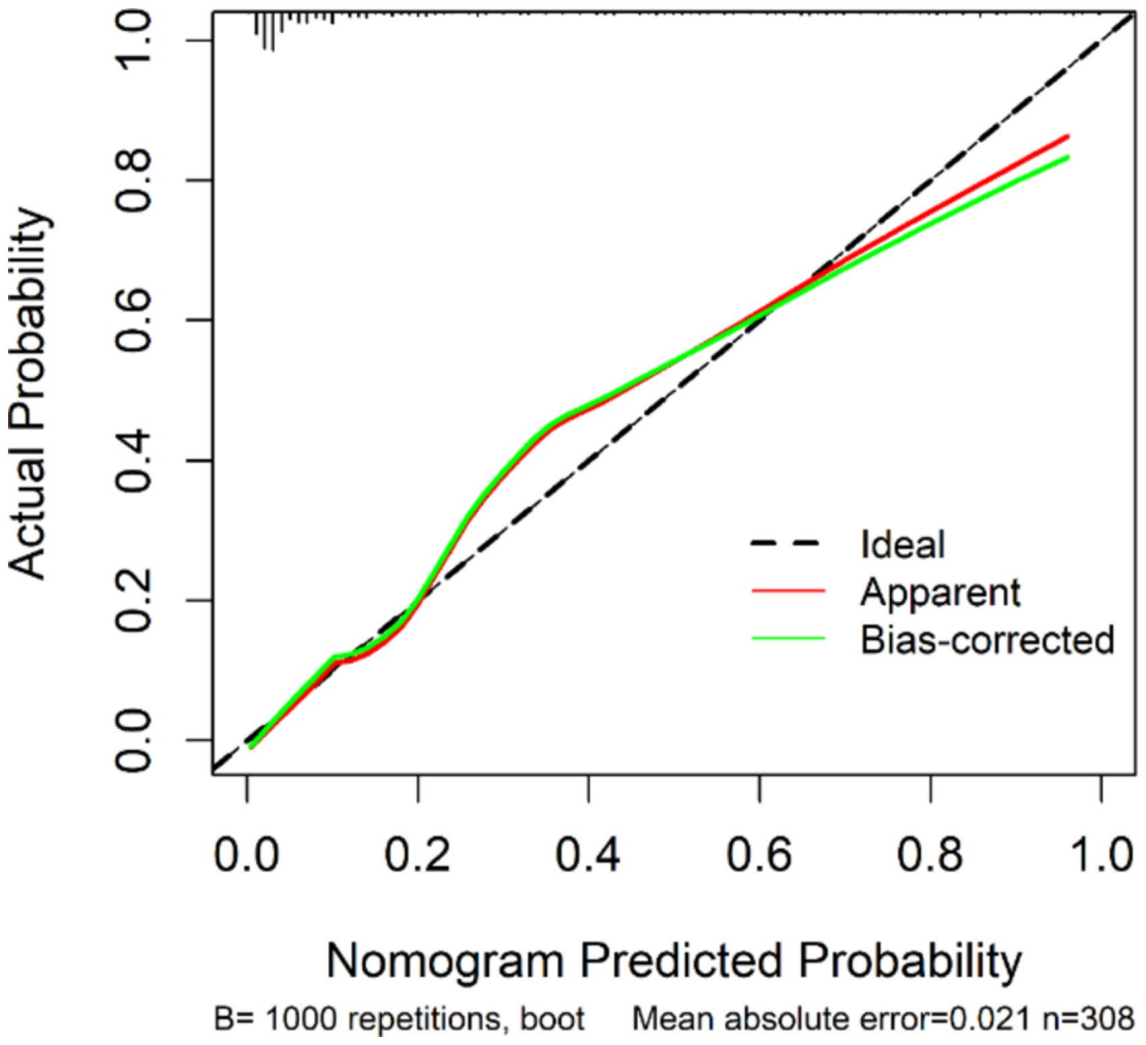
Calibration curve of MLRM.

Decision curve analysis (Figure 8) revealed that the MLR model, which incorporates aneurysm morphology, aneurysm wall characteristics, and parent artery wall features, provided more significant clinical benefit in decision-making than the other models.

## 4 Discussion

The PHASES and ELAPSS scores are widely used for assessing the risk of IA rupture, focusing on a limited set of clinical and morphological factors. HR-VWI is recognized as a superior diagnostic tool for intracranial vascular pathologies, including aneurysm diagnosis (20, 21). As research into IA imaging progresses, evidence suggests that the pathological changes in the aneurysm wall and parent artery, identified by HR-VWI, are associated with IA rupture. This study expanded upon traditional clinical and morphological characteristics by incorporating aneurysm and parent artery features from HR-VWI. We identified irregular aneurysm shape, MW, CR_stalk_, and PCR_stalk_ as independent predictors of IA rupture through univariate and multivariate logistic regression analyses. Based on these four variables, an MLRM was developed.

In this study, we assessed aneurysm size in three dimensions—NW, MW, and MH—and identified MW as an independent risk factor for IA rupture. While previous research has used various morphological factors to differentiate IA statuses, aneurysm size remains a key factor in guiding treatment decisions (22). Akio et al. (23) found that aneurysms larger than 7 mm are at higher risk of SAH, with an annual rupture rate of 0.95% for larger aneurysms. Our findings confirm that aneurysm size is linked to rupture risk, with the MW significantly larger in the ruptured group compared to unruptured groups. This is likely due to the increased pressure on the aneurysm wall as it enlarges. However, some studies have found that about one-third of patients with aneurysmal SAH have IAs smaller than 5 mm (24). In our study, the median MW in the ruptured group was 4.31 [3.30, 6.89], indicating that the risk of rupture for smaller aneurysms should not be overlooked.

Consistent with previous research, this study identified irregular aneurysm shape as a significant predictor of IA rupture. A study of 713 IAs demonstrated that irregular shape is associated with rupture, independent of size, location, and clinical characteristics (25). Another study focusing on mirror IAs at the middle cerebral artery bifurcation indicates that size and shape were the only predictive factors for rupture (26). Additionally, irregular aneurysm shape is an independent risk factor for rebleeding in patients with SAH (27). Irregularity in IAs often manifests as lobulated or multilobed structures with uneven surfaces or small protrusions linked to hemodynamic factors (28). The swirling blood flow within the aneurysm sac can cause uneven wall stress, resulting in local protrusions. As stress on these protrusions increases, the aneurysm wall thins due to continuous tension, making these weak points more susceptible to rupture under sudden pressure changes.

Inflammation plays a key factor in the formation, progression, and rupture of IAs, and AWE is a reliable indicator of the inflammatory state within the aneurysm wall. Samaniego EA et al. (29) highlighted that HR-VWI enhancement is crucial for assessing IA rupture risk, especially the absence of wall enhancement, indicating low rupture risk. HR-VWI is increasingly seen as an inflammatory biomarker. Previous studies, such as the review by Lehman VT et al. (30), compared HR-VWI images of different types of aneurysms with conventional MRI images. They concluded that HR-VWI characterizes aneurysm walls in greater in-vivo detail than was previously possible and may complement other forms of luminal imaging. Based on its excellent imaging features for aneurysms and parent arteries, we chose to extract morphological parameters and measure enhancement on HR-VWI images for model construction. Our study conducted qualitative and quantitative analyses of AWE in ruptured intracranial aneurysms (RIAs) and unruptured intracranial aneurysms (UIAs), confirming previous findings (16). The qualitative analysis demonstrated that 78.4% of RIAs exhibited focal or ring-like enhancement compared to 30.0% of UIAs (*p* < 0.05), suggesting that AWE helps identify ruptured and unstable IAs. However, since focal and ring-like AWE can also occur in UIAs, and qualitative distinction is challenging, quantitative analysis is more helpful in assessing rupture risk. This study used two quantitative measurement methods for AWE assessment: one using the right frontal lobe SI as a reference for WEI and another using pituitary enhancement SI as a reference for CR_stalk_. Results found that WEI and CR_stalk_ were significantly higher in the ruptured group, with CR_stalk_ identified as an independent risk factor for IA rupture. Compared to WEI, CR_stalk_ measurement is more straightforward, reproducible, and offers higher sensitivity and specificity, making it more suitable for quantitative analysis (16). Furthermore, building on the correlation between parent artery wall enhancement within 3 mm of the aneurysm neck and AWE (11), this study included PAWE in the analyses. It was found that both PWEI and PCR_stalk_ were significantly higher in the ruptured group, with PCR_stalk_ identified as an independent risk factor for IA rupture.

An MLRM incorporating the four identified variables—irregular aneurysm shape, MW, CR_stalk_, and PCR_stalk_—achieved an area under the curve (AUC) of 0.814. This model demonstrated superior performance with 91.7% sensitivity, 70.4% specificity, and 76.5% accuracy, notably outperforming the PHASES (AUC = 0.654) and ELAPSS (AUC = 0.642) scores. The visual nomogram derived from this model exhibited strong discrimination and calibration, offering a comprehensive tool for predicting IA rupture and guiding personalized risk management and treatment. Compared to models based on CTA and DSA that utilized multivariable logistic regression and reported AUCs of 0.80 and 0.771 (31–33), respectively, our HR-VWI-based model achieved an AUC of 0.814, indicating enhanced performance in leveraging HR-VWI for aneurysm assessment. However, machine learning and deep learning models from these studies, which integrate clinical, morphological, and radiomic features, reported even higher AUCs, such as 0.878 and 0.929. These discrepancies underscore distinct methodological approaches: while our study emphasizes HR-VWI-specific features, these prior studies highlight the importance of multi-modality integration to enhance predictive accuracy. To advance this field, integrating HR-VWI-based radiomic data into machine learning and deep learning frameworks can leverage the strengths of high-resolution imaging and multi-feature analysis. This integration may offer deeper insights into aneurysm characteristics and enhance clinical applicability. Future research should explore the complementary value of different imaging modalities and analytical techniques to develop more robust and clinically relevant models.

There are areas for improvement in this study. First, long-term follow-up of patients was not conducted, and the dynamic observation of their aneurysm development had a certain impact on the reliability of the results. Second, the dataset used for model training was derived from a single center, which may introduce selection bias. Third, the limited data volume is insufficient to support artificial intelligence modeling. Future research should explore intelligent prediction methods for assessing aneurysm risk using multicenter, big data approaches.

## 5 Conclusion

This study identified MW, aneurysm shape (irregularity), CR_stalk,_ and PCR_stalk_ as independent risk factors for IA rupture. A visual nomogram based on HR-VWI was developed and outperformed the conventional PHASES and ELAPSS scores. This model provides a valuable tool for non-invasive, non-radiative, comprehensive, and precise assessment of IA rupture risk.

## Data Availability

The raw data supporting the conclusions of this article will be made available by the authors, without undue reservation.

## Glossary

AR: Aspect ratio
AUC: Area under curve
AWE: Aneurysm wall enhancement
AWT: Aneurysm wall thickness
CE-HR-VWI: Contrast-enhanced high-resolution vessel wall imaging
CI: Confidence interval
CR_stalk_: Contrast ratio of the aneurysm wall against the pituitary stalk
DCA: Decision curve analysis
FOV: Field of view
HR-VWI: High-resolution vessel wall imaging
IAs: Intracranial aneurysms
ICC: Intraclass correlation coefficient
MH: Maximum height
MLRM: Multivariate logistic regression model
MW: Maximum width
NPR: Neck-to-parent ratio
NW: Neck width
PAD: Parent artery diameter
PCR_stalk_: Contrast ratio of the parent artery wall against the pituitary stalk
PAWT: Parent artery wall thickness
PWEI: Parent artery wall enhancement index
ROC: Receiver operating characteristic
ROI: Region of interest
SAH: Subarachnoid hemorrhage
SI - Signal intensity
SR: Size ratio
TOF: Time-of-flight
VISTA: Volume isotropic turbo spin echo acquisition
WEI: Wall enhancement index
